# Characteristics of Randomized Trials Published in Latin America and the Caribbean According to Funding Source

**DOI:** 10.1371/journal.pone.0056410

**Published:** 2013-02-13

**Authors:** Ludovic Reveiz, Stephanie Sangalang, Demian Glujovsky, Carlos E. Pinzon, Claudia Asenjo Lobos, Marcela Cortes, Martin Cañón, Ariel Bardach, Xavier Bonfill

**Affiliations:** 1 Health Systems Based on Primary Health Care, Pan American Health Organization (PAHO), Washington D.C., United States of America; 2 Argentine Cochrane Centre - Institute for Clinical Effectiveness and Health Policy (IECS), Buenos Aires, Argentina; 3 Instituto de Investigaciones, Fundación Universitaria Sanitas, Bogotá, Colombia; 4 Centro Rehabilitación Oral Avanzada e Implantología (CRAI) Universidad de Concepción (Centro Adherido Chileno de la Red Cochrane Iberoamericana), Santiago, Chile; 5 Chilean Branch of the Iberoamerican Cochrane Network, Universidad Católica de la Santísima Concepción, Santiago, Chile; 6 Facultad de Medicina, Fundación Universitaria Sanitas, Bogotá, Colombia; 7 Iberoamerican Cochrane Centre. Sant Pau Biomedical Research Institute (IIB-Sant Pau), The Biomedical Research Centre Network for Epidemiology and Public Health (CIBERESP) - Universitat Autònoma de Barcelona, Barcelona, Spain; University of Ottawa, Canada

## Abstract

**Introduction:**

Few studies have assessed the nature and quality of randomized controlled trials (RCTs) in Latin America and the Caribbean (LAC).

**Methods and Findings:**

The aims of this systematic review are to evaluate the characteristics (including the risk of bias assessment) of RCT conducted in LAC according to funding source. A review of RCTs published in 2010 in which the author's affiliation was from LAC was performed in PubMed and LILACS. Two reviewers independently extracted data and assessed the risk of bias. The primary outcomes were risk of bias assessment and funding source. A total of 1,695 references were found in PubMed and LILACS databases, of which 526 were RCTs (N = 73.513 participants). English was the dominant publication language (93%) and most of the RCTs were published in non-LAC journals (84.2%). Only five of the 19 identified countries accounted for nearly 95% of all RCTs conducted in the region (Brazil 70.9%, Mexico 10.1%, Argentina 5.9%, Colombia 3.8%, and Chile 3.4%). Few RCTs covered priority areas related with Millennium Development Goals like maternal health (6.7%) or high priority infectious diseases (3.8%). Regarding children, 3.6% and 0.4% RCT evaluated nutrition and diarrhea interventions respectively but none pneumonia. As a comparison, aesthetic and sport related interventions account for 4.6% of all trials. A random sample of RCTs (n = 358) was assessed for funding source: exclusively public (33.8%); private (e.g. pharmaceutical company) (15.3%); other (e.g. mixed, NGO) (15.1%); no funding (35.8%). Overall assessments for risk of bias showed no statistically significant differences between RCTs and type of funding source. Statistically significant differences favoring private and others type of funding was found when assessing trial registration and conflict of interest reporting.

**Conclusion:**

Findings of this study could be used to provide more direction for future research to facilitate innovation, improve health outcomes or address priority health problems.

## Introduction

The science and technology divide between developed and developing countries continues to widen as research and the benefits of innovation are disproportionately generated and used by developed countries compared to developing countries. Although more investments in research and development (R+D) have recently been made in some Latin America and the Caribbean (LAC) countries like Brazil, no country has achieved the ‘Commission on Health Research for Development’ recommended goal of allocating at least 2% of national health budgets health research and building health research capacity [1]. Science in Latin America has experienced growth in the past decade (i.e. the rate of world's scientific publications increased from 1.8% in 1991–1995 to 3.4% in 1999–2003; the growth in the numbers of Master Degrees and PhDs) and indicates a considerable effort to consolidate national systems of Science and Technology in the Region and to promote the development of R+D. One of the underlying causes of the growth in Latin American scientific activity is the increase in the number of Master degrees and Doctorates in science in some of the countries of the region. However, rates of scientific production and capacities are still low as compared to other regions and the relative impact of Latin American science is still below world averages [2], [3].

As previously pointed out by Hermes-Lima et al, there is a need to establish effective policies to increase not only the numbers of publications and highly educated researchers, but also to increase the competitiveness in terms of the quality and visibility of Latin American sciences [2]. Research and development production in LAC is frequently deterred by constrained resources, lack of capacities, difficulties to accede information and brain-drain, among others [2], [3].

Also, few studies in the region have assessed the nature and quality of scientific investigations, particularly randomized controlled trials (RCTs) in the region [4], [5]. As a result, it is uncertain whether or not RCTs in LAC are conducted in a way that facilitates innovation, improves health outcomes, and produces socioeconomic benefits for all, especially for marginalized groups whose access to innovative products and services has historically been limited [6]–[9].

Although previous studies have assessed RCTs in countries located in different regions like Asia [10], [11], Europe [12], and Sub-Saharan Africa [13], few have assessed RCTs in LAC [4], [5]. The lack of knowledge about the characteristics of the studies including their quality and the source of funding are problematic for many reasons. At the individual level, it could have negative effects on producers and users of research such as ambiguity related to how to conduct research and ensure that standards are being met; difficulty in securing and maintaining consistent funding sources; and the lack of certainty regarding the validity findings. At the population level, the lack of knowledge on research characteristics is challenging because of negative effects on health and innovation systems such as unequal resource allocation; ineffective research prioritization; decreased research capacity; lack of incentive to promote R+D in school curricula and in workplaces; decreased compensation for research professionals (giving them more reason to migrate and contribute to the brain drain).

In addition, decision makers may not have the information needed to develop health policies that are based on sound evidence. If findings are based on low quality study designs, then they may or may not be reliable or valid. Possible consequences of this lack of information are increased rates of mortality and morbidity. The health and social consequences of this lack of evidence are significant considering that in 2000, for example, cancer mortality cost the public $115.8 billion [14]. On the other hand, findings of RCTs have been effectively applied for decreasing mortality rates [15].

National research agendas frequently differ from private ones and there is an ongoing debate about which areas of research should receive public funding and how much and regarding differential relevance of national versus global sources and public versus private versus other sources of funding. At the global level, the funding is even more problematic because LAC is considered a middle income region despite the prevalence of health disparities. Compared to all regions, except Africa, LAC has the highest maternal mortality ratio (MMR) and a higher under-five year's mortality rate [16]. The discrepancy is also a matter of human rights and social justice, because those groups with the greatest needs are often excluded from R+D investments. The paradox is best described by the “10/90 Gap,” in which less than 10% of annual research investments ($160.3 billion in 2005) [17] are used to address the diseases that affect more than 90% of the world's poorest people [18].

The purpose of this study was to evaluate the characteristics (including the risk of bias assessment) of RCTs reports published by authors affiliated to institutions(s) located in LAC according to funding source.

## Methods

### 1. Study Design

A review of RCTs in LAC published in 2010 was conducted using PubMed and LILACS. We focused on the 2010 year because it provides a description of recent published RCT. Databases were searched for articles that listed a first author or contact author whose affiliate institution was in LAC (see supporting information: Appendix S1: search strategy). A previous publication evaluating the prevalence of trial registration and comparing methodological characteristics between registered and non-registered RCT used the same set of trials [19].

### 2. Sample

#### 2.1 Eligibility

Inclusion criteria: 1) published in print or published “ahead” electronically between January 1 and December 31, 2010; 2) published as an original article; 3) LAC-affiliated authors identified by the search strategy; 4) involved human subjects or clusters of human subjects; 5) random assignment used to place participants into different study groups (explicitly using the word “*random*” or variations thereof); 6) conducted in at least one LAC country site.

Exclusion criteria were: 1) identified duplicate RCTs; 2) published as secondary article in which study methods were not fully reported in this publication, but were detailed in another publication; 3) sampling of human body parts (e.g. randomization of extracted teeth, biopsies).

#### 2.2 Selection of RCT reports

A structured search for identifying RCTs was conducted; the search strategy involved PubMed (Appendix S1) and LILACS (using filter as proposed by BIREME's webpage http://lilacs.bvsalud.org/) databases. Specific filters for country and language were not used. Titles and abstracts from references were identified and screened. When there was uncertainty, the full paper was obtained to determine inclusion. Screening and selection criteria were applied in duplicate.

#### 2.3 Data extraction

Two reviewers extracted data from full papers of abstracts about RCTs that were deemed appropriate for inclusion. They assessed the risk of bias (RoB) using an instrument described in the Cochrane Collaboration Handbook [20]. The RoB tool consists of six domains (sequence generation, allocation concealment, blinding, incomplete outcome data, selective outcome reporting and ‘other issues’) for assessing the risk of bias of an RCT. RCT were evaluated independently by two reviewers and disagreements were resolved by a third reviewer. The assessment instrument was evaluated during a pilot test by all assessors. Taking into account the amount of work and resources required to assess the risk of bias of all studies, a random sample (random sequence was generated using Excel) was analyzed.

### 3. Outcomes

Descriptive variables were: publication language, first author or contact author affiliation country, intervention type, follow up duration, scope, study setting (country, multinational, multi-center), sample size, sex (of subject), ethical considerations, conflict of interest and trial registration. Prospective registration was defined as trials that were registered before the first participant is recruited. A number of priority topics related with the Millennium Development Goals were also explored and included maternal health, high priority infectious diseases (HIV, malaria, tuberculosis, neglected diseases) and a number of priority problems related with children (nutrition, pneumonia, diarrhea) [21].

Analytic variables were: risk of bias measured by the instrument proposed by the Cochrane Collaboration [20] and funding source. The Cochrane Collaboration methods for risk assessment comprise 6 domains, including randomized sequence generation; allocation concealment; masking of participants, personnel, and outcome assessors; incomplete outcome data reporting; selective outcome reporting; and other sources of bias. We classified each domain according to risk of bias, as high, low, or unclear. Funding sources were categorized as “public” (governmental either form LAC or from other region), “private”, “others” (private-public patterns such as the Global Fund), “unclear” (funders could not be classified as either public or/and private) or “not reported”. WebPages of identified sources of funding were searched to determine their type and avoid misclassification. Studies with different funding sources were compared for a number of study characteristics.

### 4. Criteria for establishing categories

RCTs were categorized according to funding source: public, private, or other. This categorization was based on findings from previous studies about corporate influence on health, which noted the importance of identifying research funding sources [22]–[24]. Two categories were used for comparison (received exclusively or partially public funding versus others).

### 5. Statistical analysis

A descriptive analysis of all evaluated articles was performed using SPSS version 17.0 (SPSS Inc. Chicago, IL, USA). Although no formal sample size was calculated, we *a priori* decided to assess at least 2/3 of all trials to assess the risk of bias. In order to assess differences according to the Risk of Bias instrument, the number and proportion of reports describing each item was calculated. All risk of bias domains were assessed for differences according to funding. Chi square (X^2^) statistics and 2-tailed Fisher exact tests were used to examine the significance of the association between categorical variables. The Holm-Bonferroni method for multiple testing procedures was used [25]. A difference was considered to be statistically significant when P≤0.05. PRISMA Checklist is available as supporting information (see Appendix S2).

## Results

### 1. Characteristics of the total number of trials

**Figure 1:**
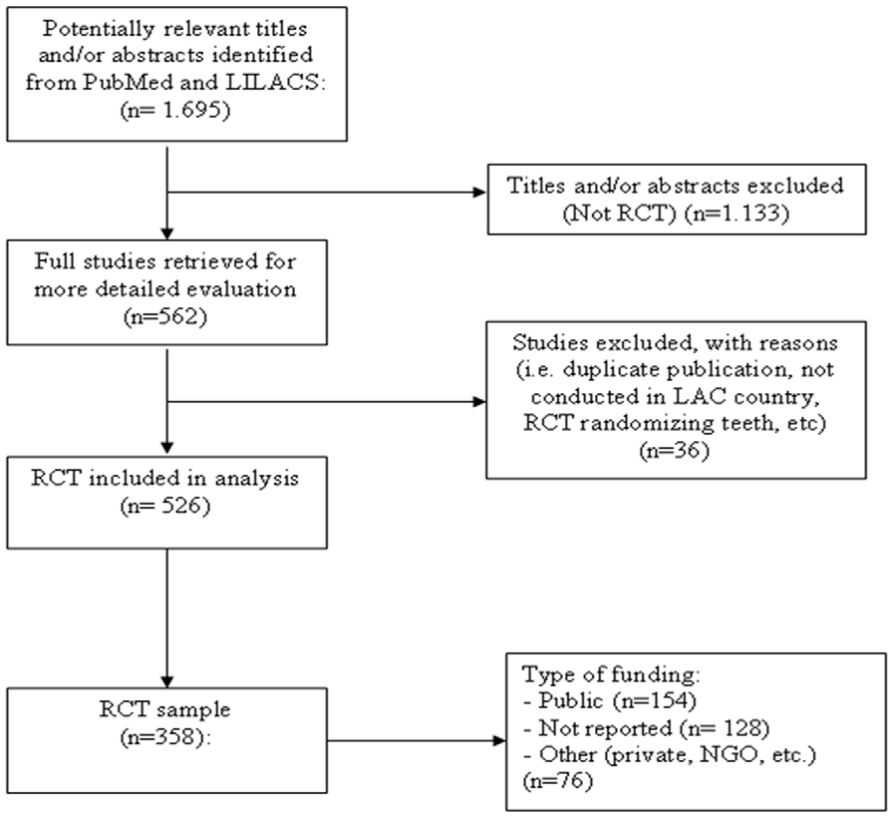
Flow diagram of the process of identifying and including studies.

A total of 1,695 references were found in the PubMed and LILACS databases, of which 526 were RCTs (N = 73,513 participants) that were included and analyzed in this study. An average of 139.8 participants (SD = 284.53) were recruited in each trial. English was the dominant publication language (93%), followed by Spanish (3.4%) and Portuguese (2.9%). Most of the RCTs were published in non-LAC journals (whose mailing address is not in the LAC region) (84.2%). Authors' affiliate institutions were in 19 LAC countries. However, just five of the 19 countries accounted for nearly 95% of all RCTs conducted in the region. Brazil (70.9%) represented the greatest majority, followed by Mexico (10.1%), Argentina (5.9%), Colombia (3.8%), and Chile (3.4%). Few RCTs covered high priority areas related with Millennium Development Goals like maternal health (6.7%) or high priority infectious diseases (3.8%). Regarding children, 19 (3.6%) and 2 (0.4%) RCT evaluated nutrition and diarrhea interventions respectively but none pneumonia; for comparison, aesthetic and sport related interventions account for 4.6% of all trials. Nine studies (1.7%) were cluster RCTs and 20 (3.8%) focused on pharmacology (i.e. bioequivalence, pharmacokinetics, pharmacodynamics, bioavailability). A flow diagram of the process for identifying and selecting studies for analysis is shown in Figure 1.

### 2. Characteristics of the sub-sample

A comparison sample of RCTs (n = 358) was assessed for select characteristics:

#### 2.1 Funding was reported as follows

Exclusively public (33.8%), private (e.g. pharmaceutical company) (15.3%), other (e.g. mixed, NGO) (15.1%); source of funding was not reported in more than one third of RCTs (35.8%). The most frequent public funding sources were in Brazil: Fundação de Amparo à Pesquisa do Estado de São Paulo (FAPESP), Coordenação de Aperfeiçoamento de Pessoal de Nível Superior (CAPES), and Conselho Nacional de Desenvolvimento Científico e Tecnológico (CNPq); United States health agency such as the National Institute of Health and US AID were reported in nine of the 358 RCT. Statistically significant differences between RCTs and funding source were detected in the following characteristics: study setting, trial registration, and conflict of interest reporting. These will be explained below in more detail along with other RCT characteristics (Table 1).

**Table 1:** Characteristics of RCTs according to reported type of funding*.

Characteristic	Public (received exclusively or partially public funding) (N = 154)	Others** (N = 76)	Significance
Multinational	5 (3.3%)	18 (23.7%)	P = 0.001
Multicenter	21 (13.6%)	27 (35.5%)	P = 0.0001
Infectious diseases such as neglected, HIV, Malaria, Tuberculosis	14 (9.1%)	2 (2.6%)	Ns
No report of ERC approval	7 (4.6%)	3 (4.0%)	Ns
No report of informed consent	9 (5.8%)	5 (6.6%)	Ns
Children	27 (17.5%)	12 (15.8%)	Ns
Conflict of interest reporting	8 (5.2%)	27 (35.5%)	P = 0001
Trial registration	32 (20.8%)	27 (35.5%)	P = 0.0164
Prospective trial registration	6 (3.9%)	9 (11.8%)	P = 0.0224
Sample size >100 participants	37 (24%)	27 (35.5%)	Ns
Follow-up longer than 6 months	35 (22.7%)	23 (30.3%)	Ns
Pharmacological intervention	79 (51.3%)	41 (54.0%)	Ns

* RCTs not reporting the type of funding were excluded from the analysis.

** Includes private, NGO.

Abbreviations ERC: ethics research committee; NS: not significant.

#### 2.2 Risk of bias

Overall assessments for risk of bias showed no statistically significant differences (P>0.05) according to funding source when analyzing publicly funded RCTs (received exclusively or partially public funding). Publicly funded RCTs had the following overall assessment for risk of bias scores: low (24.7%), unclear (58.4%), and high (16.9%). Non- publicly funded RCTs had the following overall assessment for risk of bias scores: low (36.9%), unclear (52.6%), and high (10.5%). Areas where publicly and non-publicly funded RCTs differed significantly were in the “high” and “unclear” categories of the Free of Selective Reporting assessment. Selective outcome reporting was judged to be at low risk of bias in 22.1% of publicly funded RCTs, compared to 7.9% of non-publicly funded trials (P<0.05). Unclear-scoring exclusively publicly funded RCTs had a 76.6% risk for bias compared to unclear-scoring non-exclusively publicly funded RCTs which had 89.55% risk (P<0.05). No significant differences were found for the other risk of bias domains when comparing studies with different sources of funding (Table 2).

**Table 2:** Risk of bias assessment of RCTs according to reported type of funding.

Risk of bias domain	Risk of bias assessment	Public (received exclusively or partially public funding) (N = 154)	Others** (N = 76)	Significance
*Sequence generation*	Low	71 (46.1%)	42 (55.3%)	Ns
*Sequence generation*	Unclear	80 (51.9%)	32 (42.1%)	Ns
*Sequence generation*	High	3 (2.0%)	2 (2.6%)	Ns
*Allocation concealment*	Low	49 (31.8%)	34 (44.4%)	Ns
*Allocation concealment*	Unclear	93 (60.4%)	38 (50%)	Ns
*Allocation concealment*	High	12 (7.8%)	4 (5.3%)	Ns
*Blinding of participants, personnel, and outcome assessors*	Low	70 (45.5%)	35 (46.1%)	Ns
*Blinding of participants, personnel, and outcome assessors*	Unclear	62 (40.2%)	34 (44.7%)	Ns
*Blinding of participants, personnel, and outcome assessors*	High	22 (14.3%)	7 (9.2%)	Ns
*Incomplete outcome data and Withdrawals*	Low	113 (73.4%)	55 (72.4%)	Ns
*Incomplete outcome data and Withdrawals*	Unclear	23 (14.9%)	16 (21.1%)	Ns
*Incomplete outcome data and Withdrawals*	High	18 (11.7%)	5 (6.6%)	Ns
*Free of selective reporting*	Low	34 (22.1%)	6 (7.9%)	P<0.05
*Free of selective reporting*	Unclear	118 (76.6%)	68 (89.5%)	P<0.05
*Free of selective reporting*	High	2 (1.3%)	2 (2.6%)	Ns
*Other sources of bias*	Low	95 (61.7%)	49 (64.5%)	Ns
*Other sources of bias*	Unclear	42 (27.3%)	22 (28.9%)	Ns
*Other sources of bias*	High	17 (11.0%)	5 (6.6%)	Ns
*Overall assessment*	Low	38 (24.7%)	28 (36.9%)	Ns
*Overall assessment*	Unclear	90 (58.4%)	40 (52.6%)	Ns
*Overall assessment*	High	26 (16.9%)	8 (10.5%)	Ns

#### 2.3 Trial registration

Statistically significant differences (P = 0.0164) were detected in trial registration status (including both prospective and retrospective registration) depending on funding source: 20.8% of publicly funded RCTs compared to 35.5% of non-publicly funded RCTs were registered. Similarly, significant differences (P = 0.0224) were detected in prospective trial registration status depending on funding source: 3.9% of publicly funded RCTs compared to 11.8% of non-publicly funded RCTs.

#### 2.4 Scope, setting, and subjects

The scope of RCTs covered the following specialty areas: dentistry (10.6%), gynecology-obstetrics (8.7%), anesthesia (5.6%), cardiology (5.9%), and infectious diseases (5.5%) (Table 3). A higher non-significant proportion of studies related with high priority infectious diseases were conducted with exclusive public funding when compared to exclusive private funding (6.82% vs. 0%) (P = NS).

**Table 3:** Specialty areas covered by assessed RCTs .

Specialty area	Number	Percentage
Acupuncture	2	0.6%
Aesthetic Medicine	1	0.3%
Allergology	2	0.6%
Anesthesia	20	5.6%
Cardiology	21	5.9%
Cardiovascular surgery	1	0.3%
Craneofacial surgery	1	0.3%
Dentistry	38	10.6%
Dermatology	5	1.4%
Endocrinology	16	4.5%
Family medicine	4	1.1%
Gastroenterology	10	2.8%
Genetics disease	1	0.3%
Gynecology/Obstetrics	31	8.7%
Hemodynamic	1	0.3%
Human Resources	1	0.3%
Immunology	5	1.4%
Infectious diseases	20	5.6%
Intensive care medicine	3	0.8%
Internal Medicine	1	0.3%
Nephrology	7	2.0%
Neumonology	1	0.3%
Neurology	6	1.7%
Neuroscience	1	0.3%
Nursing	2	0.6%
Nutrition	4	1.1%
Occupational Therapy	1	0.3%
Oncology	8	2.2%
Ophthalmology	10	2.8%
Orthopedics	5	1.4%
Otolaryngology	2	0.6%
Palliative medicine	1	0.3%
Pediatrics	19	5.3%
Pharmacology	13	3.6%
Physical Therapy	10	2.8%
Health promotion	1	0.3%
Psychiatry	16	4.5%
Psychobiology	2	0.6%
Public Health	1	0.3%
Pulmonology	11	3.1%
Rehabilitation	3	0.8%
Rheumatology	4	1.1%
Sleep medicine	3	0.8%
Sports medicine	19	5.3%
Surgery	13	3.6%
Transplantation medicine	1	0.3%
Urogynecological	2	0.6%
Urology	8	2.2%
**Total**	**358**	**100.0%**

The setting of most RCTs was one country and one center only. Multinational (7.7%) and multi-center (12.1%) RCTs were less common, although differences between exclusively publicly and non-exclusively publicly funded RCTs that were multinational (P<0.001) and multi-center (P<0.0001) were statistically significant; non-publicly funded trials were more often multinational and multicenter. About one-tenth of RCTs (10.5%) were co-authored by foreigners (most frequently from the USA, Canada and European countries). More than half of all participants (57.4%) were women and 17% of RCTs involved children less than 18 years old. More than ¾ of RCTs (75.4%) involved both men and women while 5.2% and 19.4% of RCTs recruited exclusively men or women respectively. Sex/gender analysis (e.g. subgroup, multivariate) was performed in 6.8% of RCTs.

#### 2.5 Intervention and follow up

The most frequent intervention types involved drugs (48.3%), followed by procedures (23.2%), behavior modification, education, counseling (10.1%), vaccines (3.1%), and devices (2.8%). A significant difference (p = 0.002) was found in the proportion of drug trials when comparing exclusive private funding (79.2%) to public funding (46.2%).

The most frequent follow up period, accounting for nearly 3/4 (72%) of all RCTs was short term: less than one month (38%) and 1–6 months (39%). Only 15 (4.2%) RCTs had a follow up period longer than three years.

#### 2.6 Ethics and conflicts of interest

Nearly all RCTs reported an informed consent process (92.2%) and even more reported approval by an ethics review committee (94.7%). The presence of conflict of interest statements differed significantly (P<0.001) between publicly and non-publicly funded RCTs. Although 42.2% of RCTs contained no declaration whatsoever, those that did were mostly non- publicly funded (35.5%). Less than half (46.9%) of all RCTs contained an author's declaration of no conflict of interest. Of note, 10.9% of RCTs affirmatively declared conflict of interest.

## Discussion

### Main findings

Assessing characteristics of RCTs conducted in LAC has provided greater insight into the state of health research in the area, highlighting both areas of progress and areas of disparity. First, Brazilian researchers were by far the dominant actors, producing more than 70% of all RCTs in LAC in our sample. Of note, the population of Brazil represents roughly one third of the total population of LAC [26]. Second, the selection of health topics for RCTs does not completely correspond to regional health priorities [21] as described in the Health of the Americas 2007 publication [27]. Maternal and children health and infectious neglected diseases-HIV/AIDS, Tuberculosis and malaria, have been identified as high priority health issues to address in LAC. They are also Millennium Development Goals (MDGs) defined by the United Nations [16]. Yet they are not being studied in RCTs conducted in LAC as frequently as expected [28]. A previous study analyzing the relationship between the global burden of disease and RCTs conducted in Latin America also found a poor correlation [29]. Although those high priority health areas frequently have well known efficacious treatments, more research, including RCTs is needed. For example, a recent study found research gaps in maternal mortality that still need to be addressed [30].

Third, the nature of RCT funding in LAC is sometimes ambiguous or not reported. When it is, most RCTs are found to be publicly funded. However, RCTs funded by private or mixed sources are characterized differently (often reporting more information compared to publically funded RCTs) in terms of study setting, trial registration status, and conflict of interest declaration. Differences between publicly and non-publicly funded RCTs in these key areas were found to be statistically significant. We presume that private companies could have more resources to perform multinational studies and also should comply with international regulations and standards. An important number of studies particularly from Brazil, were funded by public sector in the context of Master and Doctorate programs that tend to be developed by single centers and with limited resources. On the other hand, one recent study, which reviewed all records of clinical trials submitted for review and possible approval by the National Institute of Health (INS) of Peru between 1995 and 2012 found that the transnational pharmaceutical industry was the main sponsor in 87.1% of 1255 approved trials [31].

In addition, the reporting of RCTs is inconsistent. While the amount of reporting about ethics and informed consent was high, the amount of reporting about other important information remains lacking. For example, not enough reporting is done about funding source and declaration of conflict of interest. We also found that the methodology of RCTs is variable. For example, it is uncertain whether appropriate measures are taken to decrease bias or limit threats to validity. Though no statistically significant differences were found in RCT funding source and risk of bias, the most frequent score for all RCTs assessed was “unclear,” regardless of assessment type. It is difficult to conclude if the differences found were due to poor reporting or different methods/characteristics. Unfortunately, few studies have evaluated the quality of RCT in LAC and it is difficult to establish if deficiencies are due to incomplete report and/or to methodologically poor designs; to the best of our knowledge, this is the first study assessing the risk of bias related to funding source in the region [32], [33].

Finally, differences between study subjects are not always accounted for. More women than men subjects, and more adults than children (less than 18 years old), were involved in RCTs. Few RCTs report information about gender analysis. Therefore, it is uncertain whether or not findings from RCTs could be generalized to the rest of the population. However, it has to be noted that the relevance of these findings depends on each specific research question.

### Health policy implications

Findings of this study could be used to provide more direction for future research, especially to encourage strong research methods, prospective trial registration, better reporting, and targeting research at priority topics for LAC. Further exploration of why certain elements were significantly different between publicly and non-publicly funded trials and how that impacts how research findings are used and how studies are conducted in the future is needed. It is also essential to improve research standards and explore the consequences of the majority of trials being assessed at unclear risk of bias. Strengthening research systems is also a key component of the Pan American Health Organization Policy on Research for Health aimed at fostering best practices and enhanced standards for research [34].

Implications for health policy include: implementing guidelines for monitoring and evaluating RCT reporting in LAC; developing strategies to improve adherence to international reporting standards (such as the Equator Network); promoting intra-regional, South-South collaboration through knowledge sharing, networking, participating in conferences, and co-writing articles for publication. Another study that provided a geographic overview of clinical cancer research indicates that multinational collaboration is increasing [35]. Other health policy implications include: creating reliable funding streams for strengthening national health research systems and addressing regional health research priorities; promoting early registration of RCTs. Ultimately, strategies to address these issues could have the following implications for health policy: 1) to facilitate the translation of research activity into tangible outputs of innovation (e.g. products, services, therapies, patents, publications) and 2) to promote the development and implementation of policy that is based on sound research evidence. The impact of LAC science is still below world averages and indicates the need to establish effective policies to enhance competitiveness in terms of quality and international recognition [2], [3].

Although progress has been made in several areas, many gaps in knowledge remain. These gaps, if not addressed promptly and effectively, can become problem areas with severe consequences for the health and socioeconomic development of LAC countries. Perhaps the most serious consequence is the decreased capacity to conduct research and translate knowledge into innovation [36]. This hurts not only research producers and users but also many marginalized groups who are in desperate need of access to basic care, never mind innovative products and services.

### Strengths and limitations

The search was restricted to only two databases and one calendar year. A major limitation of geographic filters is that the search strategy (authors from LAC) only captures the affiliation of the main author or the contact author of the study. Neither LILACS nor PubMed allow having access to all author's affiliation. Consequently, we only had access to published RCTs in which the available affiliation was declared from LAC. The institutional affiliation does not necessarily dictate the geographical location of a RCT; however we only included RCT that were conducted in at least one LAC country site. In addition, the search strategy used to identify RCT may have missed some studies. As a result, findings may not be representative of all RCTs published in LAC. We based results on a structured search strategy to identify articles and we included two reviewers to assess and extract data, with a third reviewer available to resolve any conflicts. Also, our analysis was based on the report of RCTs; thus we only had access to what authors chose to write, or not write and the article may not necessarily be a complete picture of conduct of the trial. The study used explicit inclusion and exclusion criteria and applied a formal measurement tool to assess risk of bias. Ultimately, the strength of this study lies in its unique contribution to the field. Previously, little was known about the characteristics of RCT in LAC.

### Conclusion

Some differences between publicly and non-publicly funded RCTs were found in clinical research for trial registration, ethic issues, conflict of interest reporting and trial settings among others.

## Supporting Information

Appendix S1 **Search strategy S1.**
(DOC)

Appendix S2 **PRISMA Checklist 2009.**
(DOC)
