# Supplementary material for: Characteristics of Randomized Trials Published in Latin America and the Caribbean According to Funding Source
Source: PLoS One. 2013 Feb 13;8(2):e56410. doi: 10.1371/journal.pone.0056410 (PMC3572054; doi:10.1371/journal.pone.0056410)
Supplement: Appendix S1 — Search strategy S1. (DOC) [file pone.0056410.s001.doc]

**S1 Appendix: Search strategy**

((randomized controlled trial [pt] OR controlled clinical trial [pt] OR randomized [tiab]

OR placebo [tiab] OR randomly [tiab]) NOT (animals [mh] NOT (humans [mh] AND

animals [mh]))) AND (Latin america* [ad] OR South America* [ad] OR central America*

[ad] OR carribbean* [ad] OR Anguilla [ad] OR Antigua and Barbuda [ad] OR Argentina

[ad] OR Aruba [ad] OR Bahamas [ad] OR Barbados [ad] OR Belize [ad] OR Bermuda

[ad] OR Bolivia* [ad] OR Brazil* [ad] OR brasil* [ad] OR British Virgin Islands [ad]

OR Cayman Islands [ad] OR Chile* [ad] OR Colombia* [ad] OR Costa Rica [ad] OR

Cuba [ad] OR Dominica [ad] OR Dominican Republic [ad] OR El Salvador [ad] OR

Ecuador [ad] OR French Guiana [ad] OR Grenada [ad] OR Guadalupe [ad] OR

Guatemala [ad] OR Guyana [ad] OR Haiti [ad] OR Honduras [ad] OR Jamaica* [ad] OR

Martinique [ad] OR Mexico [ad] OR Montserrat [ad] OR Netherlands Antilles [ad] OR

Nicaragua [ad] OR Panama [ad] OR Paraguay [ad] OR Peru [ad] OR Puerto Rico [ad]

OR Saint Kitts and Nevis [ad] OR Saint Lucia [ad] OR Saint Vincent and the Grenadines

[ad] OR Suriname [ad] OR Trinidad and Tobago [ad] OR Uruguay* [ad] OR Venezuela

[ad] OR el Valle [ad] OR Saint John's [ad] OR Buenos Aires [ad] OR Oranjestad [ad] OR

Nassau [ad] OR Bridgetown [ad] OR Belmopán [ad] OR sucre [ad] OR la paz [ad] OR

Brasilia [ad] OR sao paulo [ad] OR rio de janeiro [ad] OR belo horizonte [ad] OR Maceió

[ad] OR manaus [ad] OR Goiânia [ad] OR belem [ad] OR porto alegre [ad] OR

Florianópolis [ad] OR George Town [ad] OR santiago [ad] OR valparaiso [ad] OR

Concepción [ad] OR temuco [ad] OR bogota [ad] OR cali [ad] OR medellin [ad] OR

barranquilla [ad] OR san jose [ad] OR la habana [ad] OR Santo Domingo [ad] OR San

Salvador [ad] OR Quito [ad] OR guayaquil [ad] OR Saint George's [ad] OR port au

prince [ad] OR Tegucigalpa [ad] OR Kingston [ad] OR Willemstad [ad] OR managua

[ad] OR asuncion [ad] OR lima [ad] OR San Juan [ad] OR Santo Domingo [ad] OR

Kingstown [ad] OR Marigot [ad] OR Castries [ad] OR Paramaribo [ad] OR Montevideo

[ad] OR Caracas [ad] OR Road Town [ad] OR Mexicali [ad] OR Tuxtla Gutiérrez [ad]

OR chihuahua [ad] OR Guanajuato [ad] OR guadalajara [ad] OR toluca [ad] OR morelia

[ad] OR Monterrey [ad] OR puebla [ad] OR argentinean [ad] OR mexican [ad] OR

bolive* [ad] OR costaric* [ad])
